# Relationship between physical exercise and college students’ social adaptation: the chain mediating role of self-esteem and peer attachment

**DOI:** 10.3389/fpsyg.2024.1453675

**Published:** 2024-10-29

**Authors:** Zehui Zhou, Kelei Guo, Siqiang Guo, Lang Chen

**Affiliations:** School of Physical Education and Health, Zhaoqing University, Zhaoqing, China

**Keywords:** college students, physical exercise, social adaptation, self-esteem, peer attachment, mental health, mediating role

## Abstract

**Objective:**

Physical exercise is an important way for college students to keep healthy, and social adaptation is an important part of college students’ mental health. Therefore, this study explores strategies to enhance college students’ social adaptation from the perspective of physical exercise, examining the correlation between physical exercise and college students’ social adaptation, and delving into the roles of self-esteem and peer attachment in this relationship.

**Methods:**

A stratified cluster sampling method was used to collect data from 809 college students at Zhaoqing University (average age 19.88 ± 1.22, of whom 399 were male and 410 were female) using the physical exercise scale, college students’ social adaptation scale, self-esteem scale, and peer attachment scale. For data analysis, Pearson correlation analysis, structural equation modeling, and bias-corrected percentile bootstrap methods were sequentially performed.

**Results:**

(1) Physical exercise was positively correlated with college students’ social adaptation (*r* = 0.58, *p* < 0.01), and the direct path between physical exercise and college students’ social adaptation was significant (*β* = 0.28, *p* < 0.01, CI[0.22, 0.33]); (2) Physical exercise was positively correlated with self-esteem (*β* = 0.56, *p* < 0.01, CI[0.50, 0.62]) and peer attachment (*β* = 0.18, *p* < 0.01, CI[0.11, 0.26]); self-esteem was positively correlated with peer attachment (*β* = 0.36, *p* < 0.01, CI[0.28, 0.43]) and college students’ social adaptation (β = 0.43, p < 0.01, CI[0.37, 0.49]); peer attachment was positively correlated with college students’ social adaptation (*β* = 0.18, *p* < 0.01, CI[0.12, 0.23]); (3) The relationship between physical exercise and social adaptation was not only mediated independently by self-esteem and peer attachment, but also indirectly by the same two factors in a chain reaction.

**Conclusion:**

Physical exercise can not only directly predict college students’ social adaptation, but also indirectly predict college students’ social adaptation through the independent mediation and chain mediation of self-esteem and peer attachment. It reveals that we should combine more important physical exercise with mental health education for students.

## Introduction

With the rapid development of science and technology and cultural diversification in China, the social adaptability of university students is receiving unprecedented attention. Social adaptation is crucial for academic success and future careers of university students. However, current research often fails to comprehensively consider the various factors affecting college students’ social adaptive capacity, such as the residential setting, the academic setting, the features of the population, and the psychological traits of each individual. Nevertheless, social adaptation refers to the ability to adjust one’s behavior and attitudes according to the social living environment, achieving a state of coordination and integration between the individual and the environment (Zhu, 2011). The “Medium and Long-Term Youth Development Plan (2016–2025)” issued by the State Council of China explicitly states that school education should support college students in engaging in various extracurricular and off-campus activities, providing targeted guidance for their social integration. College students should learn to live and survive, actively understand and adapt to society. As a part of the future social force, college students need to better integrate into society and meet social needs, and through physical exercise can reduce anxiety and pressure, improve self-control ability and social support. Therefore, in order to provide theoretical direction for increasing social adaptation through physical exercise, this study intends to investigate how physical exercise impacts college students’ social adaptation and whether self-esteem and peer attachment influence this link.

### Physical exercise and social adaptation

Physical exercise is a kind of activity that enhances physical fitness, improves health and promotes mental health through physical activity, which can be carried out in free time, with the main purpose of health, and with a certain intensity, frequency and duration (Song, 2001). College students should actively participate in physical exercise, as it not only enhances their physical and mental health but is also a basic requirement from educational institutions during higher education. Different categories of college students’ physical exercise have varying degrees of impact on their social adaptation abilities, showing a significant correlation (Xiao, 2007). Students who engage in physical exercise exhibit stronger social abilities than those who do not, and those involved in team sports demonstrate even stronger social adaptation abilities (Wu, 2018). Studies indicate that physical exercise has a contributory effect on the social adaptability of university students, and that the highest and good social adaptability indices of university students in the collective ball sports and the small ball games against each other across the nets are positively correlated with the increase in the number of times per week of physical exercise and intensity of exercise loads (Cui, 2013). Moderate physical exercise can effectively enhance college students’ social adaptation abilities (Ma, 2017), further indicating that maintaining appropriate physical exercise time and intensity significantly improves social adaptation abilities (Chen and Sto, 2021). Research data show that non-physical exercise students had poor social adaptation, physical exercise students who participated in group sports were more socially adapted, and senior students were also socially adapted, and physical exercise had a positive impact on the social adaptation of university students (Ji and Zheng, 2021). Therefore, we propose Hypothesis 1: Physical exercise is positively correlated with college students’ social adaptation.

### The mediating role of self-esteem

Self-esteem is an individual’s emotional response to their physical characteristics, personality, and self-worth, which is an important element of personality psychology, affecting the level of mental health, personality character traits, and personality psychological traits of an individual, and is therefore subjected to research on self-esteem by psychologists from different perspectives (He and Xu, 2002; McAdams et al., 2021). Numerous studies have shown that physical exercise can improve self-esteem levels and has a significant impact (Feng and Zhao, 2012; Wang, 2016). Research indicates that that basketball, badminton, and aerobics exercise have different degrees of moderating effects on college students with low levels of self-esteem, and aerobics has a significant improvement on the self-esteem of both male and female college students, and basketball and badminton are more significant on the self-esteem levels of male college students (Xue and Xu, 2006; Xu and Yao, 2001). Experimental studies have shown that hockey, running, and team sports effectively improve physical self-esteem (Edwards et al., 2005; Wang and Cai, 2008). Other studies indicate that high-intensity physical exercise significantly increases physical self-esteem levels compared to low-intensity exercise (Zhang and Sun, 2009), and the longer college students engage in physical exercise, the higher their self-esteem levels (Guo, 2016). Physical exercise has a direct and significant positive impact on an individual’s physical self-esteem level (Guo et al., 2023).

The well-adapted dimension of college students’ self-esteem is significantly positively correlated with social adaptation, while the poorly adapted dimension is significantly negatively correlated (Jiang and Yu, 2015). There is a significant correlation between vocational students’ self-esteem and social adaptation; individuals with high self-esteem have better social adaptation (Yu, 2020). Studies have shown that improving college students’ self-esteem plays an important role in social adaptation, with a significant positive correlation between self-esteem and social adaptation (Fan, 2011). Self-esteem significantly positively predicts college students’ learning adaptation (Chen et al., 2008). This implies a correlation between college students’ self-esteem and social adaptation: the higher the self-esteem level, the higher the social adaptation level (Xin, 2016). Liu and Chen (2020) further confirm this viewpoint, demonstrating a significant direct effect of college students’ self-esteem on social adaptation, showing a significant positive correlation. Based on the above, we propose Hypothesis 2: Self-esteem mediates the relationship between physical exercise and social adaptation.

### The mediating role of peer attachment

Peer attachment refers to the emotional bond established between individuals and their peers, characterized by feelings of intimacy, warmth, and mutual support (Liu and Chen, 2020). This relationship, often marked by similar age characteristics or psychological health, plays an important role in the physical and mental health development and social adaptation of college students. There is a significant positive correlation between physical exercise and peer attachment. Physical exercise among college students can significantly predict positive peer attachment, with more solid and harmonious peer relationships established through physical exercise (Chen and Yu, 2015). Studies indicate that physical exercise significantly predicts peer attachment, and active participation in physical exercise can enhance peer attachment, gain peer support and recognition, and easily establish stable peer relationships (Ma et al., 2022). Other research shows significant differences in peer attachment among students with varying degrees of physical exercise participation. The longer college students participate in physical exercise, the higher the level of peer support they receive, which provides satisfaction and happiness during activities with peers, thereby enhancing peer relationships and increasing the degree of peer attachment (Li et al., 2023). Hence, physical exercise promotes the development of peer attachment.

There is a significant positive correlation between peer attachment and social adaptation among college students, and college students’ mutual trust and positive communication can help improve their social adaptation ability (Liu and Chen, 2020). Research by Dou Fen et al. shows a close relationship between college students’ peer attachment and social adaptation, with a significant positive correlation. Peer attachment promotes physical and mental health, reducing the likelihood of depression (Dou et al., 2018). Peer attachment significantly predicts social adaptation positively, with college students having good peer relationships exhibiting more prosocial behavior (Li et al., 2023). Peer attachment significantly positively predicts social adaptation, with good peer relationships enhancing a sense of belonging and life satisfaction (Cui, 2023). Based on the above, we propose Hypothesis 3: Peer attachment mediates the relationship between physical exercise and social adaptation.

### Chain mediating role of self-esteem and peer attachment

**Figure 1:**
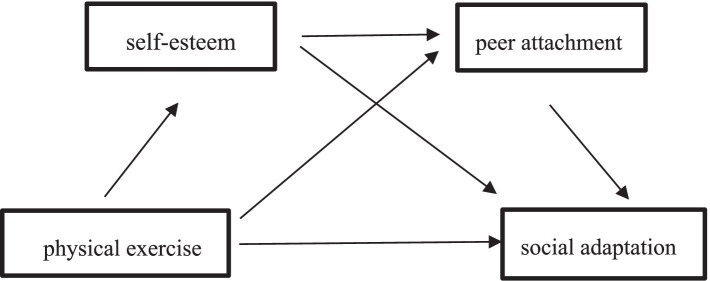
Research on mediation model.

Self-esteem is the realization of self-worth and positive self-evaluation (Chen and Wang, 2006), while peer attachment reflects college students’ individual cognition, emotion and social adaptability. Research has found that the higher the level of self-esteem of college students, the higher their peer attachment (Liu and Chen, 2020). Low self-esteem tends to lead to conflict and betrayal with peers, while high self-esteem is strongly associated with positive peer relationships and high-quality friendships (Huang, 2017). There is a positive correlation between self-esteem and peer attachment, where individuals with high self-esteem hold a positive attitude toward themselves, have better confidence, and are skilled at establishing good peer relationships (Lü and Qiu, 2020). Given the previously discussed positive correlations between physical exercise and self-esteem, peer attachment, and the positive correlations between self-esteem, peer attachment, and social adaptation, we propose Hypothesis 4: Self-esteem and peer attachment serve as chain mediators between physical exercise and social adaptation (Figure 1).

## Materials and methods

### Procedure and participants

Using stratified cluster sampling, a survey was conducted among enrolled college students. Five classes were randomly selected from each grade of each department, and the survey was administered at the class level. Questionnaire distribution focused on 14–27 November 2023. A total of 916 questionnaires were distributed, and after excluding those with incomplete basic information or patterned responses, 809 valid questionnaires were recovered, resulting in an effective recovery rate of 88.32%. The participants were aged 18–22 years (mean age = 19.88 ± 1.22), including 399 males and 410 females, with 223 freshmen, 198 sophomores, 204 juniors, and 184 seniors.

In accordance with the Declaration of Helsinki, this study received approval from the Research Ethics Committee of Zhaoqing University (No. 2024014). All participants were informed of the purpose and nature of the study and signed informed consent forms. Participation was voluntary, and confidentiality was assured. The primary examiners were trained physical education students. During the testing process, consent from both teachers and students was obtained.

### Measures and instruments

#### Physical exercise scale

The Physical Exercise Scale developed by Chen et al. (2006) and revised by Wu (2016) was used. The scale includes two dimensions: commitment to physical exercise and adherence to physical exercise, with four items each, making a total of eight items (e.g., “I rarely interrupt my physical exercise and can persist in it for a long time”). Responses were rated on a 5-point Likert scale ranging from “strongly disagree” to “strongly agree,” scoring 1–5 points respectively, with the total score representing the participant’s level of physical exercise. The higher the total score, the higher the level of physical activity. This scale has been proven to have high applicability among Chinese college students (Jiang et al., 2018). In this study, the internal consistency reliability coefficient *α* was 0.693.

#### Self-esteem scale

Self-esteem was measured using the Rosenberg Self-Esteem Scale (Rosenberg, 1965), revised by Wang et al. (1999) for measuring college students’ self-esteem. The scale contains 10 items (e.g., “I feel that I am a person of worth.”), scored on a 4-point scale (1 = strongly disagree, 4 = strongly agree), with a theoretical score range of 10–40 points, where higher scores indicate higher levels of self-esteem. Previous studies have shown that this scale has good applicability among Chinese college students (Xie et al., 2023). In this study, the Cronbach’s *α* coefficient was 0.893.

#### Peer attachment scale

Peer attachment was measured using the peer attachment subscale of the Inventory of Parent and Peer Attachment (IPPA) developed by Armsden and Greenberg (1987), which consists of 25 items (e.g., “I like to get my friends’ opinions on things that are important to me.”), scored on a 5-point scale ranging from “strongly disagree” to “strongly agree,” scoring 1–5 points, respectively. The scale was revised into Chinese by Song (2004), with good reliability and validity. The scale includes three factors: peer trust, peer communication, and peer alienation. This scale has been proven to have high applicability among Chinese college students (Liu and Chen, 2020). In this study, the Cronbach’s *α* coefficient was 0.800.

#### Social adaptation scale

The College Students’ Social Adaptation Scale developed by Fang (2008) was used to evaluate college students’ social adaptation. This scale includes five dimensions: learning adaptation with 6 items (e.g., “When I encounter difficulties in learning, I can generally solve them independently”), interpersonal adaptation with 3 items (e.g., “I do not feel uncomfortable in crowded places”), psychological adaptation with 4 items (e.g., “My classmates are excellent, but I think I am also a valuable person”), environmental adaptation with 5 items (e.g., “I find it not difficult to adapt to the school’s living conditions”), and future adaptation with 5 items (e.g., “I pay close attention to future social development trends to avoid falling behind”). The scale consists of a total of 23 items. Responses were rated on a 5-point scale from “strongly disagree” to “strongly agree,” scoring 1–5 points respectively, with higher total scores indicating stronger social adaptation abilities. This scale has been proven to have high applicability among Chinese college students (Cheng and Zhao, 2019). In this study, the Cronbach’s *α* coefficient was 0.866.

### Statistical analyses

Descriptive analysis, Pearson correlation analysis, and chain mediation effect testing using the Process component were conducted with SPSS 26.0. According to the non-parametric percentile bootstrap method proposed by Hayes (2018), the mediation effect test was conducted using PROCESS (Version 3.3) macro model 6 with 5,000 bootstrap samples and a 95% confidence interval (CI) for demographic variables. Differences were considered statistically significant at *p* < 0.05.

## Results

### Common method deviation test

Since all study subjects were from the same university, there might be common method bias affecting the statistical results. Therefore, Harman’s single-factor test was conducted on the collected data. The results showed that 15 factors were generated, and the variance explained by the first factor was 17.27%, which is less than the critical value of 40%. Thus, it was concluded that there was no serious common method bias.

### Descriptive statistics and correlation analysis

As shown in Table 1, the correlation coefficients among physical exercise, self-esteem, peer attachment, and social adaptation were all statistically significant. Correlation analysis revealed that physical exercise was significantly positively correlated with self-esteem, peer attachment, and social adaptation; self-esteem was significantly positively correlated with peer attachment and social adaptation; and peer attachment was significantly positively correlated with social adaptation. These findings provided preliminary support for our hypotheses.

**Table 1:** Means, standard deviations, and correlations among variables.

Variable	*M*	SD	1	2	3	4
1. Physical exercise	23.06	4.86	1			
2. Self-esteem	26.88	5.12	0.56**	1		
3. Peer attachment	85.11	10.61	0.38**	0.46**	1	
4. Social adaptation	68.16	13.57	0.58**	0.67**	0.48**	1

*N* = 809; **p < 0.01.

### Mediation effect analysis

**Figure 2:**
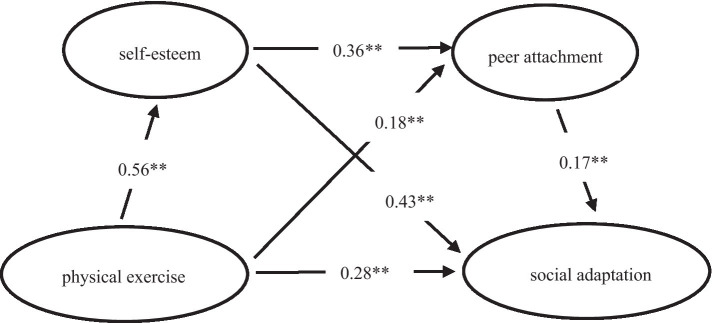
Chain mediation model of self-esteem and peer attachment between physical exercise and social adaptation. ** Not only self-esteem and peer attachment play an independent mediating role between physical exercise and social adaptation, but also self-esteem and peer attachment play a chain mediating role between physical exercise and social adaptation of college students.

First, we examined the direct path between physical exercise and social adaptation. The results showed that the direct path between physical exercise and social adaptation was significant, *β* = 0.28, *p* < 0.01, CI [0.22, 0.33], thus confirming Hypothesis 1. Next, we examined the mediating roles of self-esteem and peer attachment between physical exercise and social adaptation (see Figure 2). The results indicated that physical exercise was positively correlated with self-esteem, *β* = 0.56, *p* < 0.01, CI [0.50, 0.62]; self-esteem was positively correlated with social adaptation, *β* = 0.43, *p* < 0.01, CI [0.37, 0.49], confirming Hypothesis 2. Physical exercise was positively correlated with peer attachment, β = 0.18, *p* < 0.01, CI [0.11, 0.26]; peer attachment was positively correlated with social adaptation, β = 0.18, *p* < 0.01, CI [0.12, 0.23], confirming Hypothesis 3. Self-esteem was positively correlated with peer attachment, β = 0.36, *p* < 0.01, CI [0.28, 0.43], confirming Hypothesis 4. The mediation effect test results (see Table 2) showed that the chain mediation effect of self-esteem and peer attachment was significant, and the simple mediation effects of self-esteem and peer attachment were also significant.

**Table 2:** Mediation effect and effect size.

Path	Effect size	Proportion of total effect	95% confidence interval	
			Lower limit	Upper limit
Physical exercise → self-esteem → social adaptation	0.24	0.24/0.31 = 77.42%	0.20	0.28
Physical Exercise → peer attachment → social adaptation	0.03	0.03/0.31 = 9.68%	0.02	0.05
Physical Exercise → self-esteem → peer attachment → social adaptation	0.04	0.04/0.31 = 12.90%	0.02	0.05
Total effect	0.31		0.26	0.34

The values presented in Figure 2 are the values of *β* which are tested by stepwise regression method, β is the standardized coefficient.

## Discussion

This study explores the relationship between physical exercise and college students’ social adaptation, as well as the mediating effects between self-esteem and peer attachment. The results show that physical exercise can not only positively affect social adaptation, but also indirectly affect social adaptability through the independent mediating effect of self-esteem and peer attachment. Furthermore, physical exercise also affect social adaptation through the chain mediation of self-esteem and peer attachment. Besides, in the practical teaching of improving college students’ social adaption, in addition to encouraging physical exercise, special emphasis should be made to the promotion of self-esteem and peer attachment.

### The relationship between physical exercise and social adaptation

The results of this study show a positive correlation between physical exercise and social adaptation, indicating that physical exercise can enhance social adaptation abilities. Hu and Wang (2017) believe that physical exercise can directly promote mental health, improve cognitive abilities and self-esteem levels, obtain social support, and improve interpersonal relationships and social adaptation abilities. Their research indicates a high correlation between physical exercise and social adaptation, where appropriate physical exercise can effectively enhance college students’ social adaptation abilities (Ma, 2017). Personality psychology theory suggests that social adaptation and personality adaptation are interrelated, and the relatively stable emotions and consistent psychological traits exhibited by individuals toward society help them better adapt to society, improving their quality of life and social adaptation abilities. During college, appropriate physical exercise effectively promotes mental health and enhances social abilities. By enriching extracurricular activities through physical exercise and improving physical fitness, college students’ overall quality is enhanced. This process helps regulate emotions and strengthens willpower, thereby promoting mental health development and enhancing social adaptation abilities (Ji and Zheng, 2021). However, due to the lack of a unified research consensus on the time, frequency, and intensity of physical exercise, further evidence is needed to support this result.

### Independent mediating effect of self-esteem

The results of this study indicate that self-esteem mediates the relationship between physical exercise and social adaptation. This is consistent with previous research evidence that shows a positive correlation between physical exercise and self-esteem (Guo et al., 2023; Guo, 2023), and a positive correlation between self-esteem and social adaptation (Liu et al., 2022; Xu, 2013).

From the perspective of sports psychology, there is a close relationship between physical exercise and self-esteem. Regular participation in physical exercise has a positive impact on self-esteem, benefiting mental and physical health, improving mood, and reducing stress. Overcoming challenges during exercise can enhance self-confidence and self-efficacy, and this sense of accomplishment further boosts self-esteem. Si (2013) in the physical exercise on college students physical self-esteem and self-confidence in the measurement and evaluation of the impact of the study found that the level of self-esteem with the increase in the frequency of exercise, exercise duration, exercise intensity and significantly increased, while Wang (2016) also proved this point of view. Individuals who exercise regularly are likely to experience positive exercise experiences and gain a sense of achievement after completing planned exercise tasks. In addition, regular exercise over a long period of time not only changes body shape, but also enhances self-efficacy, which leads to a more positive evaluation of one’s own body, thus increasing overall self-esteem (Li et al., 2015). Additionally, a longitudinal study of 642 college students discovered that low self-esteem predicts social problems, while poor self-esteem itself contributes to eventual social difficulties (Crocker and Luhtanen, 2003). In contrast, positive peer response support is mentioned as a way to assist people in reaching their objectives and boosting their self-esteem (Feeney, 2004). In summary, there is a significant positive correlation between physical exercise and self-esteem (Wei, 2022). College students can improve their self-esteem levels through good exercise habits and active participation in physical exercise. There is also a significant positive correlation between self-esteem and social adaptation (Li and Wu, 2015). College students’ participation in moderate physical exercise helps enhance self-esteem, making them more proactive and communicative in social interactions, which improves their social adaptation abilities. Therefore, self-esteem mediates the relationship between physical exercise and social adaptation, confirming Hypothesis 2.

### Independent mediating effect of peer attachment

The results of this study indicate that peer attachment mediates the relationship between physical exercise and social adaptation. This is consistent with previous research evidence that shows a positive correlation between physical exercise and peer attachment (Li et al., 2022), and a positive correlation between peer attachment and social adaptation (Liu and Chen, 2020).

Physical exercise positively predicts peer attachment (Hu, 2022). Physical exercise often involves group activities that require cooperation among peers, mutual trust, and peer attachment, which is the foundation of empathy development. It emphasizes the close emotional bond between individuals and their peers. People who frequently participate in sports activities have higher security in peer attachment. There is a significant positive correlation between physical exercise and peer attachment among university students, and regular participation in physical activity is conducive to building trust, enhancing peer relationships, obtaining peer acceptance and support, and facilitating the experience of interpersonal interaction (Li et al., 2023). College students with poor peer attachment find it difficult to establish intimate relationships with their peers, leading to negative emotions and further negative evaluations of themselves and the world, increasing the risk of depression, which is detrimental to mental health development and social abilities (Ye et al., 2019). The research results indicate that peer attachment plays a significant mediating role between physical exercise and social adaptation. This finding is important for explaining the relationship between the two and exploring the internal mechanisms at play. Therefore, it is necessary to pay attention to college students’ peer attachment relationships and consider them as an important pillar for improving their social adaptation abilities.

### Chain mediating effect of self-esteem and peer attachment

This study also found that self-esteem and peer attachment play a chain mediating role between physical exercise and social adaptation. In other words, physical exercise can indirectly affect social adaptation through the chain mediating effect of self-esteem and peer attachment. Individuals with high self-esteem are less likely to have negative evaluations of themselves, are less likely to fall into negative thinking, and are more likely to take the initiative to communicate positively with their peers, and their peer attachment is good, whereas individuals with low self-esteem have negative perceptions and an avoidance mentality, and find it difficult to be accepting and tolerant of others, which results in poor peer attachment (Wang and Liu, 2024). Additionally, Wu and Zhang (2023) indicated that college students with high self-esteem levels have a positive evaluation of peer attachment, easily achieve peer mutual assistance, confidently face difficulties, and establish self-affirmation. In contrast, those with low self-esteem are more likely to perceive the negative impact of peer pressure, magnify their own flaws in interactions, and doubt their self-worth. Thus, higher self-esteem levels correspond to higher levels of peer attachment (Liu et al., 2016). This study found a positive correlation between self-esteem and peer attachment. Moreover, the chain mediating effect of self-esteem and peer attachment between physical exercise and social adaptation was significant, helping to understand the connections between these factors more deeply and revealing the mediating effect between physical exercise and social adaptation. Therefore, in the process of enhancing college students’ social adaptation, it is necessary to consider the combined effects of self-esteem and peer attachment. In summary, self-esteem and peer attachment play a chain mediating role between physical exercise and social adaptation, confirming Hypothesis 4.

### Practical implications

This study explored the relationship between physical exercise and college students’ social adaptation abilities, deeply analyzing the mechanism between physical exercise and social adaptation, expanding the research field of college students’ social adaptation. From the perspective of physical exercise, the study provides a possible solution path for addressing college students’ social adaptation issues and offers empirical support for society and educational departments to mobilize college students to actively participate in physical exercise. College students’ social adaptation is a focus of national and societal concern, and guiding college students toward healthy growth and helping them establish correct life values has important practical significance. First, since there is a positive correlation between physical exercise and social adaptation, colleges should foster a positive campus sports culture, organize diverse sports activities, improve physical education curricula, and conduct targeted physical education teaching to encourage students to actively participate in physical exercise. Second, self-esteem and peer attachment are important elements of college students’ social adaptation, directly influencing their social adaptation abilities and combining with peer attachment factors to produce effects. This suggests that families, schools, and society should emphasize enhancing college students’ self-esteem and peer attachment to further improve their social adaptation abilities.

### Limitations and perspectives

This study is based on existing research and related theoretical foundations, but due to the limitations of questionnaire methods and cross-sectional research, it is difficult to infer causal relationships between variables. Future research could use longitudinal tracking or experimental intervention studies. Additionally, this study focused on exploring the relationship mechanisms of social adaptation among participants from a single university. Future research could conduct tracking studies on larger samples. Finally, although this study considered the mediating effects of self-esteem and peer attachment between physical exercise and social adaptation, further examination of other important variables closely related to social adaptation, such as subjective well-being and basic psychological needs, is necessary to comprehensively explain the relationship model between physical exercise and social adaptation.

## Conclusion

(1) Physical exercise was significantly and positively related to social adaptation; (2) Self-esteem was an independent mediator between physical exercise and social adaptation; (3) Peer attachment was an independent mediator between physical exercise and social adaptation; (4) Self-esteem and peer attachment were chained mediators between physical exercise and social adaptation. It is proposed that in addition to focusing on improving college students’ attitudes and behaviors toward physical exercise, mental health interventions and promotion should also address their self-esteem and peer attachment.

## Data Availability

The raw data supporting the conclusions of this article will be made available by the authors, without undue reservation.
